# HPV in Pregnancy: Implications for Screening, Vaccination, and Maternal–Fetal Health

**DOI:** 10.1155/jp/1466858

**Published:** 2026-03-18

**Authors:** Suman Kumar, Swati, Swati Salila, Akanksha Raj, Pratima Gupta, Neha Sharad

**Affiliations:** ^1^ Department of Microbiology, All India Institute of Medical Sciences, Deoghar, Jharkhand, India, aiims.edu; ^2^ Department of Obstetrics and Gynecology, All India Institute of Medical Sciences, Deoghar, Jharkhand, India, aiims.edu; ^3^ Department of Microbiology, ESIC Medical College & Hospital, Patna, Bihar, India; ^4^ Department of Obstetrics and Gynaecology, Anugrah Narayan Magadh Medical College & Hospital, Gaya, Bihar, India, unicamp.br

**Keywords:** diagnostics, HPV, HPV vaccination, pregnancy, preterm birth, vertical transmission

## Abstract

Human papillomavirus (HPV) infection is the most common sexually transmitted infection worldwide. It is highly prevalent among women of reproductive age. During pregnancy, hormonal changes and immunological modulation may promote viral persistence, thus likely increasing the risk of adverse maternal and neonatal outcomes. However, the clinical significance of HPV infection in pregnancy remains incompletely understood. This qualitative thematic analysis is aimed at evaluating the epidemiological and clinical impact of HPV during pregnancy, focusing on pathophysiological mechanisms, diagnostic challenges, obstetric and perinatal outcomes, and gaps in clinical management. A comprehensive literature search of PubMed and Scopus was conducted for peer‐reviewed original studies published in the last 10 years in English. The review followed PRISMA 2020 guidelines. Of 1667 records initially identified, 34 studies met the inclusion criteria and were included in the qualitative synthesis. Study quality was assessed using the Newcastle–Ottawa Scale. Given the heterogeneity of study designs and outcomes, a thematic narrative synthesis was performed. Four major themes emerged: (1) pregnancy‐related immunological, hormonal, and microbiome changes that facilitate HPV persistence; (2) diagnostic challenges arising from physiological cervical changes that affect cytology and colposcopy accuracy; (3) associations between maternal HPV infection and adverse outcomes such as preterm birth, premature rupture of membranes, miscarriage, low birth weight, and vertical transmission; and (4) disparities in screening and vaccination policies between high‐income countries and low‐ and middle‐income countries. While HPV is not directly teratogenic, evidence suggests it may indirectly compromise placental function and the integrity of the cervix. In conclusion, HPV infection during pregnancy is a clinically relevant concern with potential implications for both maternal and fetal health. Standardized screening strategies, improved vaccination coverage, and longitudinal studies are needed to guide evidence‐based clinical practice and policy development.

## 1. Introduction

Human papillomaviruses (HPVs) are a group of DNA viruses that exist with more than 200 different subtypes, making them the most common sexually transmitted infection in the world [1]. Many people with HPV are asymptomatic, but it can lead to serious health issues, including cancers in the anogenital area and the oropharynx. In particular, it is quite prevalent among women of childbearing age, which raises concerns. A persistent HPV infection during pregnancy might interfere with the placenta and affect the fetus′s development and could lead to complications like premature birth, miscarriage, or low birth weight. Despite how common HPV is, we still do not fully understand its implications during pregnancy. Research on this topic has been inconsistent, with studies varying widely in their design, populations, and the outcomes they measure. A lot of existing research also focuses on vaccination, studies outside of pregnancy, or how the virus spreads among men. There is a significant need for a current, comprehensive overview of research from the past 10 years to help guide clinical decisions.

Reported HPV prevalence among pregnant women ranges from 5% to 40% [2–5], with high‐risk types more persistent during pregnancy. Changes in the immune system and hormonal shifts might contribute to this persistence and could potentially speed up the progression of cervical disease. While HPV is not directly known to cause birth defects, it has been linked to negative pregnancy outcomes [4–8].

### 1.1. Relevance of the Study

To date, no recent thematic synthesis has comprehensively addressed HPV‐related maternal and fetal outcomes during pregnancy. Given how widespread HPV is and the possible repercussions for both mothers and babies, it is crucial to gather and evaluate recent evidence on this topic. This analysis seeks to provide a current overview of HPV during pregnancy, pinpoint areas where we lack knowledge, and help shape clinical practices and preventative measures.

### 1.2. Aim and Objectives

The primary aim of this review is to explore the clinical and epidemiological impact of HPV infections during pregnancy.

The objectives are as follows:

• To evaluate the pathophysiological and immunological implications of HPV during pregnancy
• To assess diagnostic challenges and maternal–fetal risks associated with HPV infection
• To identify gaps in clinical management and suggest areas for future research and policy development

## 2. Materials and Methods

This study adopted a secondary thematic analysis for a qualitative systematic review to examine HPV infections during pregnancy. The primary aim was to extract and synthesize key themes related to the clinical and epidemiological implications of HPV in the context of pregnancy.

### 2.1. Data Sources and Search Strategy

A comprehensive literature search was conducted in PubMed and Scopus. The initial search retrieved 1667 records, which were narrowed to 918 after applying a 10‐year publication filter. Screening for full‐text availability, study relevance, and adherence to inclusion criteria ultimately resulted in 34 studies included in the review.

### 2.2. Inclusion and Exclusion Criteria

Studies were included if they were as follows:

• Peer‐reviewed original research, clinical studies
• Reported at least one of the following: pathogenesis of HPV in pregnancy, clinical diagnosis, maternal or neonatal complications, or prevention/management strategies
• Published in English

Exclusion criteria were as follows:

• Studies focused on male‐only populations
• Review articles, systematic reviews, meta‐analyses, editorials, letters, or conference abstracts

### 2.3. Thematic Analysis

Data from the included studies were extracted and analyzed using a thematic framework.

Key findings were coded and grouped into four primary themes:

• Pathophysiological processes of HPV during pregnancy
• Clinical presentation and diagnostic challenges
• Obstetric and perinatal complications
• Guidelines for screening and vaccination

This framework enabled a clear categorization of heterogeneous findings, providing a structured narrative on the impact of HPV on maternal and fetal outcomes.

### 2.4. Study Selection and PRISMA Flow

The study selection process followed PRISMA 2020 guidelines. The initial 1667 records were screened for eligibility, with 918 records remaining after applying the 10‐year filter. Further exclusions were applied for studies that were irrelevant, duplicates, inaccessible in full text, or did not meet the inclusion criteria, resulting in 34 studies included in the qualitative synthesis (Table 1).

**Table 1:** Summary of key studies included in the review.

Ref	Year	Country	Study design	Population	Main findings	Notes/limitations
2	2021	China	Cross‐sectional	1200 women	HPV infection rates higher in pre‐ vs. postmenopausal women; age‐related risk patterns	Single center, no longitudinal follow‐up
3	2012	South Korea	Cross‐sectional	1000 women	HPV prevalence and genotype distribution determined using RFLP assay	Regional sample only
4	2019	India	Prospective cohort	250 pregnant women	Early pregnancy HPV prevalence; potential implications for pregnancy outcomes	Small cohort, limited follow‐up
5	2014	China	Epidemiologic	600 pregnant women	HPV prevalence and risk factors identified; some association with pregnancy complications	Retrospective analysis
6	2017	Australia	Cohort	300 pregnant women	Maternal HPV infection increases the risk of premature rupture of membranes	Abstract only (conference report)
7	2009	Brazil	Prospective cohort	200 mother–child pairs	Evidence of perinatal HPV transmission	Small sample size
8	1993	Australia	Prospective	50 mother–child pairs	Maternal–child HPV transmission demonstrated	Older study, limited genotyping
11	2023	Norway and Sweden	Mother–child cohort	2500 pregnant women	Maternal HPV associated with preterm delivery	Population‐based, limited genotype info
12	2023	Puerto Rico	Cross‐sectional	200 women	Cervical microbiota is nonoptimal regardless of HPV status	Limited to Hispanic population
13	2016	Poland	In vitro/ex vivo	Amniotic epithelial cells from 40 women	HPV infection affects defensin production; the mode of delivery may influence outcomes	Laboratory‐based; small sample
14	2020	United States	Longitudinal cohort	400 women	Cervicovaginal microbiome influences the natural history of HPV	Observational, regional cohort
15	2025	Russia	Observational cohort	100 women with recurrent pregnancy loss	Lower genital tract microbiota linked to recurrent pregnancy loss	Small sample; exploratory study
17	2025	Kazakhstan	Observational	150 women	Cervicovaginal microbiome changes with age; protective against HPV	Small sample, observational
18	2023	Italy	Prospective cohort	120 women (early pregnancy)	HPV may cause miscarriage in early gestation	Small cohort, observational
19	2021	China	Cross‐sectional	1500 women	HPV prevalence higher in pregnant vs. nonpregnant women; genotype distribution documented	Regional sample, no longitudinal outcomes
20	2021	Romania	Observational	200 pregnant women	Maternal HPV infection may negatively affect pregnancy outcomes	Limited sample, observational
21	2022	United States	Retrospective cohort	100 patients	HPV‐positive oral cancers have distinct clinical and morphologic features	Not pregnancy‐related, included for context
22	2016	Thailand	Cross‐sectional	300 pregnant women	Assessed cervical cancer screening methods; prevalence of abnormal cytology reported	Single hospital, regional sample
23	2024	Romania	Observational cohort	180 pregnant women	Pregnancy‐related precancerous cervical lesions affect gestation and fertility	Small cohort, observational
24	2018	Italy	Observational	150 pregnant women	Colposcopy is reliable during pregnancy for detecting cervical lesions	Limited sample, single center
25	2024	China	Observational cohort	200 pregnant women	HPV DNA + cytology cotesting improves screening accuracy during pregnancy	Sample size moderate, single region
26	2024	Korea	Observational cohort	120 pregnant women	Cervical intraepithelial neoplasia assessed; cytology is useful in pregnancy	Small sample, hospital‐based
27	2018	Brazil	Observational	100 miscarriage cases	HPV detected in fetal and maternal tissues from miscarriage cases	Small sample, limited generalizability
28	2013	China	Cross‐sectional	250 pregnant women	High‐risk HPV infection associated with premature rupture of membranes	Regional sample, cross‐sectional
29	2019	Australia	Retrospective cohort	400 pregnant women	Maternal HPV associated with preterm premature rupture of membranes	Retrospective design, potential confounders
30	2019	India	Observational cohort	150 early pregnancy women	HPV prevalence assessed; early pregnancy implications noted	Small sample, single region
31	2025	China	Retrospective cohort	800 reproductive‐age women	HPV genotypes linked to reproductive tract inflammation and adverse pregnancy outcomes	Retrospective design, regional cohort
32	2021	Sweden	Population‐based cohort	100,000+ women	Treated and untreated HPV infections linked to preterm delivery and neonatal mortality	Large dataset, registry‐based; potential unmeasured confounders
33	2004	United States	Longitudinal	200+ pediatric patients	Recurrent respiratory papillomatosis severity associated with HPV Types 6 and 11	Pediatric population; may not generalize to adults
34	2016	China	Observational	50 juvenile patients	HPV type determination in juvenile‐onset recurrent respiratory papillomatosis	Small sample, hospital‐based
35	2019	Australia	Data linkage	10,000+ pregnant women	HPV infection associated with intrauterine growth restriction	Observational, linkage study; limited causality
36	2022	Spain	Observational	100 neonates	Neonatal oropharyngeal HPV infection detected; maternal transmission observed	Small sample, single center

### 2.5. Risk of Bias Assessment

Given that most included studies were observational, the Newcastle–Ottawa Scale (NOS) was used to assess methodological quality. Studies were evaluated on selection, comparability, and outcome/exposure ascertainment and categorized as low, moderate, or high risk of bias. Findings are presented transparently in the synthesis.

## 3. Review

### 3.1. Pathophysiological Processes of HPV During Pregnancy

During pregnancy, the way HPV behaves in the body can change quite a bit due to the shifts in our immune system and hormone levels. These changes are necessary for the protection of the developing fetus but can inadvertently make it harder for the body to fight off infections, including HPV. Hormonal changes, especially increases in estrogen and progesterone, can actually modulate how the virus acts. They can activate certain genes in HPV that lead to more viral replication and protein production. This heightened activity can lead to more persistent infections, which can, in turn, affect pregnancy outcomes negatively [9–12].

Additionally, the balance of bacteria in the cervicovaginal area plays a role in how HPV behaves. An imbalance is often seen when harmful bacteria outnumber beneficial ones like *Lactobacillus* and can create inflammation, promoting conditions that allow the virus to stick around longer. These changes might also weaken the natural barriers that help keep the virus at bay [13–18].

Moreover, HPV can impact the integrity of the cervical tissue, raising concerns of complications such as premature rupture of membranes (PROM) and preterm delivery. Research has found that pregnant women are generally more likely to have HPV compared to those who are not pregnant, especially in women over 35, indicating a rise in susceptibility during this time [19].

Overall, the journey of pregnancy influences the way HPV operates, through a mix of immune responses, hormonal fluctuations, changes in cervical tissue, and shifts in the microbiome. Together, these factors increase the risk for ongoing infections and potential cervical issues in expectant mothers.

### 3.2. Clinical Presentation and Diagnostic Challenges

HPV infection during pregnancy can often go unnoticed since it might not show any symptoms. Sometimes, women might notice mild issues like genital warts or cervical lesions. These warts are typically caused by lower risk types of HPV, specifically Types 6 and 11. During pregnancy, they might actually grow larger because of hormonal shifts and changes in the immune system [20]. On the other hand, high‐risk HPV infections can lead to more serious cervical changes, but these often do not produce any noticeable symptoms until they reach more advanced stages. There have been some studies looking into HPV‐related changes in the mouth, but translating those findings to cervical infections in pregnant women can be tricky, as it may ignore factors that are unique to pregnancy [21].

Diagnosing HPV while pregnant can be a bit tough. The body undergoes various changes during pregnancy, including increased vascularity, increased mucus production, and tissue friability. These changes can mimic dysplasia and thus reduce the sensitivity of Pap smears, increasing the risk of false‐positive results. Colposcopy is often delayed due to potential risks, such as bleeding or uterine contractions, further complicating timely diagnosis. Despite these limitations, Pap smear remains the recommended screening test during pregnancy due to its higher sensitivity and specificity for detecting precancerous cervical lesions compared with visual inspection with acetic acid (VIA) [22–25].

HPV DNA testing offers higher sensitivity and specificity than cytology alone. Cohort studies in pregnant women show that HPV testing, alone or combined with cytology, effectively identifies high‐grade lesions, particularly those associated with HPV‐16 and HPV‐18 [25, 26]. However, the cost of HPV testing can be quite high, and since Pap smears also work well, many healthcare providers stick with cytology as the go‐to screening method during pregnancy. Additionally, HPV self‐sampling has emerged as a highly acceptable approach, which may improve screening coverage in low‐resource settings [24–26].

### 3.3. Obstetric and Perinatal Complications

HPV infection during pregnancy has been increasingly associated with adverse obstetric outcomes, including preterm birth, PROM, and miscarriage (Table 2) [26–32]. Similarly, a large Swedish cohort study by Wiik et al. found that HPV infection shortly before or during pregnancy increased the risk of preterm delivery, preterm PROM (pPROM), PROM, and neonatal complications. Women with a history of cervical intraepithelial neoplasia (CIN) treatment exhibited even higher risks, indicating that post‐CIN pregnancies should be considered high‐risk and managed accordingly [32]. These findings also support the importance of HPV vaccination programs in reproductive‐age women.

**Table 2:** Summary of adverse pregnancy‐related outcomes due to HPV.

Reference	Study type/population	Pregnancy‐related complication(s)	Key findings/notes
Cotton‐Caballero et al. [6]	Observational cohort	Premature rupture of membranes (PROM)	Maternal HPV infection increased PROM risk
Wiik et al. [11]	Mother–child cohort, Norway and Sweden	Preterm delivery	Maternal HPV associated with higher risk of preterm birth
Bruno et al. [18]	Prospective study	Early miscarriage	Suggested HPV as a potential cause of miscarriage in early gestation
de Freitas et al. [27]	Case study, miscarriage tissues	Miscarriage	Detected HPV in fetal and maternal tissues of miscarriage cases
Cho et al. [28]	Cohort study	PROM	High‐risk HPV infection associated with increased PROM risk
Caballero et al. [29]	Retrospective cohort	Preterm premature rupture of membranes (pPROM)	Maternal HPV increased risk of pPROM
Pandey et al. [30]	Prospective study, early pregnancy	HPV infection in early pregnancy	Confirms prevalence and potential implications for gestation
He et al. [31]	Retrospective cohort, reproductive‐age women	Reproductive tract inflammation, adverse pregnancy outcomes	High‐risk HPV associated with adverse outcomes, including miscarriage and inflammation
Wiik et al. [32]	Population‐based study, Sweden	Preterm delivery, neonatal mortality	Both treated and untreated HPV infections linked with preterm birth and neonatal death
Ford et al. [33]	Data‐linkage study	Intrauterine growth restriction (IUGR)	HPV infection associated with higher risk of IUGR

Maternal HPV infection may also affect neonatal outcomes, resulting in low birth weight and juvenile‐onset recurrent respiratory papillomatosis (JORRP) [23–35]. Vertical transmission during vaginal delivery has been documented. In one prospective study, HPV was detected in the oropharyngeal secretions of 58.2% of neonates at birth, though 94.3% cleared the virus within 24 months, with only 5.7% showing persistence [36]. Cesarean delivery may reduce, but does not eliminate, the risk of vertical transmission [37].

Despite these associations, most studies are limited by different diagnostic criteria, a lack of follow‐up, and underrepresentation of diverse populations, which hinder the development of standardized clinical guidelines for HPV screening and management during pregnancy.

### 3.4. Guidelines for Screening and Vaccination

The World Health Organization (WHO) has set a global strategy to eliminate cervical cancer, targeting an incidence rate below 4 cases per 100,000 women‐years. Achieving this requires meeting the 90–70–90 targets by 2030 which includes vaccinating 90% of girls against HPV by Age 15, screening 70% of women with a high‐performance test by Ages 35 and 45, and ensuring 90% of women diagnosed with cervical disease receive timely treatment. Mathematical models predict that fulfilling these targets in low‐ and lower middle‐income countries (LMICs) could reduce cervical cancer incidence by 42% by 2045 and by 97% by 2120. This would also prevent over 74 million cases and save millions of lives [38].

Despite this strategy, cervical cancer screening remains challenging in many LMICs due to low awareness, cultural barriers, financial constraints, and limited healthcare infrastructure. Key barriers include lack of knowledge, embarrassment, unsupportive family or community norms, and structural issues such as cost and geographic access [39–44]. Addressing these barriers through education, health system strengthening, and community engagement is essential to increase screening coverage and move toward global cervical cancer elimination.

In contrast, developed countries such as the United States have well‐established guidelines. In the United States, Pap smear screening is recommended every 3 years for women aged 21–65 and high‐risk HPV testing every 5 years for women aged 30–65, regardless of pregnancy status [45]. However, there are no clear guidelines for HPV screening, vaccination, and management in most LMICs, thus highlighting a critical gap in preventive care.

## 4. Discussion

This analysis looks into the complex relationship between HPV infection and pregnancy, highlighting some important aspects. HPV infection during pregnancy is not uncommon and is influenced by immunological, hormonal, and microbiome changes. These changes favor viral persistence. Although often asymptomatic, HPV can cause cervical lesions, genital warts, and adverse obstetric outcomes. Vertical transmission is possible but generally transient, and long‐term neonatal outcomes remain underresearched. Screening practices are inconsistent, particularly in LMICs, and vaccination coverage remains suboptimal. This underscores the need to assess these risks during prenatal care carefully. While there is some evidence that the virus can be passed to newborns during delivery, it usually does not remain for longer periods, with most infants clearing the virus within 2 years. However, the long‐term effects on babies—such as the risk of developing JORRP—are still not well understood, highlighting a significant research gap that needs to be addressed. In addition to these concerns, there are gaps in management and clinical guidelines.

### 4.1. Gaps in Clinical Management and Recommendations for Future Research and Policy Development

Managing HPV during pregnancy can be quite challenging due to the lack of consistent guidelines. Screening protocols exist in some regions but remain largely inaccessible, particularly in LMICs. This highlights the importance of widespread HPV vaccination as a key strategy for both women and men. Efforts should be gender‐inclusive, should focus on reducing stigma, and increase awareness about HPV, its health risks, and available preventive measures [46–49].

Although HPV vaccination is not typically recommended during pregnancy, prenatal care offers an ideal opportunity to start or complete vaccination. Vaccination during pregnancy is safe and may confer protective antibodies to infants [50–52]. Thus, improving vaccine availability and affordability is crucial. In developed countries, community pharmacies with extended hours and walk‐in availability can enhance coverage. While in LMICs, integrating HPV vaccination into national immunization programs, offering catch‐up vaccination for adults, and supporting public–private partnerships can increase access [53–55].

Long‐term follow‐up of infants born to HPV‐positive mothers is limited. Thus, understanding of potential respiratory or immunological complications, like JORRP, which remains understudied despite significant morbidity is necessary [56–58].

Given the substantial morbidity and mortality associated with HPV, evidence‐based strategies to increase vaccination coverage remain important. Routine adolescent vaccination, catch‐up vaccination for unvaccinated adults, and vaccination prior to or after pregnancy may help reduce HPV‐related disease. Public education and creating awareness are also important, as limited knowledge and misconceptions contribute to low vaccine uptake. Approaches such as community sensitization, school‐based education programs, and association with healthcare providers will lead to improved acceptance and coverage. Although HPV is highly preventable, it continues to contribute to a significant global disease burden. Vaccination remains the most effective intervention to reduce HPV‐related morbidity and mortality. Thus, expanding its uptake represents a key component of public health efforts to address HPV‐related disease.

### 4.2. Strengths and Limitations of Our Review

*Strengths*: It comprehensively covers HPV infection and its pregnancy‐related complications across multiple populations. This analysis includes studies from diverse geographic regions (Asia, Europe, the United States, and LMICs). It has focused on both maternal and neonatal outcomes. The extraction of data to include population size, study design, and main findings has improved clarity and enabled comparisons.

*Limitations*: Many studies included in the analysis are observational, thus limiting the ability to infer causality. There is heterogeneity in the study populations, methods, and HPV detection techniques. Some studies have small sample sizes, especially in specific subgroups. There is limited longitudinal data on long‐term outcomes for children exposed to maternal HPV.

### 4.3. Implications for Practice

• HPV screening during pregnancy may help identify women at risk of obstetric complications like preterm birth or miscarriage.
• Clinicians should consider maternal HPV status when managing pregnancies with unexplained complications.
• Vaccination programs remain critical, and future strategies could explore safe vaccination approaches in reproductive‐age women.
• Awareness campaigns and accessibility of screening in low‐resource settings can improve early detection and reduce pregnancy‐related risks.
• Monitoring neonates born to HPV‐positive mothers may be warranted to detect early signs of vertical transmission or juvenile‐onset papillomatosis.

## 5. Conclusion

This analysis highlights critical evidence gaps, including limited longitudinal studies, different diagnostic approaches, and insufficient follow‐up of infants exposed to HPV.

Integrating cervical screening into routine antenatal care is recommended, particularly for underscreened or high‐risk women. HPV DNA testing may enhance detection where resources are optimal. Women with persistent high‐risk infection or a history of cervical disease should be closely monitored. Antenatal care provides an opportunity for education, counseling, and assessment of vaccination status, with postpartum catch‐up vaccination representing an effective preventive measure. Expanding gender‐neutral vaccination programs, improving access to screening and vaccination in resource‐limited settings, and developing standardized, pregnancy‐specific clinical guidelines are recommended priorities. Public education and awareness initiatives targeting women of reproductive age and healthcare providers are essential to improve knowledge, reduce stigma, and enhance uptake.

Future research on longitudinal cohort studies to clarify causal relationships between maternal HPV infection and adverse pregnancy outcomes will be helpful. Also, long‐term neonatal follow‐up to assess persistent infection and complications needs to be undertaken. Coordinated clinical, research, and public health efforts are essential to optimize maternal and neonatal outcomes and reduce the global burden of HPV‐related disease.

## Funding

No funding was received for this manuscript.

## Conflicts of Interest

The authors declare no conflicts of interest.

## Data Availability

Data sharing is not applicable to this article as no datasets were generated or analyzed during the current study.
